# A New Medical Journal

**Published:** 1844-02

**Authors:** 


### A NEW MEDICAL JOURNAL.

Drs. Fenner & Hester propose publishing, in New Orleans, a quarterly journal of Medicine, the first number to be issued on the 1st of May next. Each number is to contain about 200 pages, and to consist of original communications, reports from the New Orleans hospitals and infirmaries, a periscope of practical medicine, and brief notices of recent medical literature. The city of New Orleans affords many advantages for the publication of a journal of practical medicine, and we hope the proposed undertaking may be successful in developing them. A report of the doings of the profession in that great city, could not fail to be interesting to their brethren in other parts of the world. The terms of the "New Orleans Medical Journal" are $5 per annum, payable in advance.

